# Differential Susceptibility to Hypertension Is Due to Selection during the Out-of-Africa Expansion

**DOI:** 10.1371/journal.pgen.0010082

**Published:** 2005-12-30

**Authors:** J. Hunter Young, Yen-Pei C Chang, James Dae-Ok Kim, Jean-Paul Chretien, Michael J Klag, Michael A Levine, Christopher B Ruff, Nae-Yuh Wang, Aravinda Chakravarti

**Affiliations:** 1 The Johns Hopkins University School of Medicine, Baltimore, Maryland, United States of America; 2 Division of Pediatrics, The Cleveland Clinic Foundation, Cleveland, Ohio, United States of America; University of Chicago, United States of America

## Abstract

Hypertension is a leading cause of stroke, heart disease, and kidney failure. The genetic basis of blood pressure variation is largely unknown but is likely to involve genes that influence renal salt handling and arterial vessel tone. Here we argue that susceptibility to hypertension is ancestral and that differential susceptibility to hypertension is due to differential exposure to selection pressures during the out-of-Africa expansion. The most important selection pressure was climate, which produced a latitudinal cline in heat adaptation and, therefore, hypertension susceptibility. Consistent with this hypothesis, we show that ecological variables, such as latitude, temperature, and rainfall, explain worldwide variation in heat adaptation as defined by seven functional alleles in five genes involved in blood pressure regulation. The latitudinal cline in heat adaptation is consistent worldwide and is largely unmatched by latitudinal clines in short tandem repeat markers, control single nucleotide polymorphisms, or non-functional single nucleotide polymorphisms within the five genes. In addition, we show that latitude and one of these alleles, *GNB3* (G protein β3 subunit) *825T,* account for a major portion of worldwide variation in blood pressure. These results suggest that the current epidemic of hypertension is due to exposures of the modern period interacting with ancestral susceptibility. Modern populations differ in susceptibility to these new exposures, however, such that those from hot environments are more susceptible to hypertension than populations from cold environments. This differential susceptibility is likely due to our history of adaptation to climate.

## Synopsis

High blood pressure, or hypertension, is a leading cause of stroke, heart disease, and kidney failure. Hypertension is due to factors such as older age, high salt intake, obesity, and genetic susceptibility. The genetic variants responsible for increased susceptibility to hypertension are largely unknown but are likely to include those that increase salt retention and blood vessel tone. Here the authors show that populations around the world differ in susceptibility to hypertension such that populations from hot, wet environments are more susceptible to hypertension than populations from cold environments. This differential susceptibility is likely due to selection during the human out-of-Africa expansion 50,000 to 100,000 y ago. Furthermore, the authors show that latitude and one of the genetic variants that increase susceptibility to hypertension account for a major portion of worldwide variation in blood pressure. Therefore, the current epidemic of hypertension is due to ancestral susceptibility interacting with the new risk factors of the modern period, such as high salt intake and obesity. Populations differ in susceptibility to these risk factors, however. This differential susceptibility is due to our history of adaptation to climate.

## Introduction

A majority of people in industrialized countries will develop high blood pressure or hypertension. By one estimate, 90% of middle-aged and elderly people living in the United States can expect to become hypertensive [1]. The burden is not equally shared, however. In the United States, hypertension occurs earlier, more often, and with greater severity among people of African compared to European descent [2]. A portion of this greater burden may be due to greater genetic susceptibility to hypertension in some populations. Here we argue that hypertension susceptibility is ancestral and that a portion of differential susceptibility is due to differential exposure to selection pressures during the out-of-Africa expansion.

### Hypertension Susceptibility Is Ancestral

Enhanced salt and water avidity and vascular reactivity, key components of hypertension susceptibility, may have been adaptive in our ancestral African environment characterized by a hot, wet climate and salt scarcity [3–7]. Effective heat dissipation is essential in hot environments and is achieved most efficiently through evaporative heat loss [8]. As a result, humans have an unmatched capacity to sweat, being able to achieve a sweating rate of 2 l/h [9]. Sweating, however, can lead to loss of large amounts of salt and water [8]. Large salt losses due to sweating, combined with low salt availability in tropical climates [3], made a robust salt appetite and renal sodium conservation essential for survival. In support of this hypothesis, humans and non-human primates from tropical climates have enhanced salt and water avidity [3,10–12]. Another consequence of the reliance on sweating for heat dissipation is diurnal volume depletion [13]. During periods of low blood volume, increased arterial tone and force of cardiac contraction maintain blood pressure and ensure organ perfusion. Therefore, genetic variation that enhances arterial and cardiac contractility may have conferred a survival advantage in the environmental context of early human evolution.

### Differential Susceptibility to Hypertension May Be Due to Differential Exposure to Selection Pressures during the Out-of-Africa Expansion

Originating in Africa some 100,000 to 200,000 y ago [14,15], our species has since expanded out of Africa to inhabit the rest of the world. As populations expanded out of Africa, the primary thermodynamic requirement shifted from heat dissipation to heat conservation and selection for salt and water avidity, and cardiovascular reactivity lessened. As a result, people who are adapted to colder regions have diminished vascular reactivity [16,17] and salt avidity, and as a consequence actually produce more sweat during heat stress than equatorial populations [18,19]. This difference in volume avidity and vascular reactivity is a physiologic source of differential susceptibility to hypertension and may be a consequence of differential exposure to selection pressures since the out-of-Africa expansion. In support of this hypothesis, two groups have recently demonstrated evidence of selection in two genes that influence blood pressure. *AGT* (angiotensinogen) and *CYP3A5* (cytochrome P450 3A5) have single nucleotide polymorphisms (SNPs) with functional alleles that influence salt avidity and blood pressure. For both genes the allele that increases salt avidity is the major allele among populations near the equator. By contrast, the allele that decreases salt avidity has risen to high frequency outside of Africa, probably due to selection [20,21].

Do populations differ in hypertension susceptibility? Is the difference in susceptibility due to selection? What are the implications for worldwide variation in blood pressure? In order to address these questions, we first identified functional alleles in five genes that affect volume avidity and cardiovascular reactivity. The identified genes and SNPs include *AGT* (angiotensinogen) *G-217A* and *G-6A, GNB3* (G protein β3 subunit) *C825T, ADRB2* (β2 adrenergic receptor) *G47A* and *G79C, ENaCα* (epithelial sodium channel α) *A-946G,* and *ENaCγ* (epithelial sodium channel γ) *A-173G.* These SNPs were chosen based on experimental evidence that variation at each affects gene expression or function. We then determined worldwide variation in heat adaptation as defined by prevalence of the allele that increases volume avidity or cardiovascular reactivity. Finally, we determined the effect of ecological factors and hypertension susceptibility, as measured by the *GNB3 825T* allele frequency, on worldwide variation in blood pressure.

## Results

### Heat Adaptation Is Strongly Associated with Latitude, Temperature, and Precipitation

**Figure 1:**
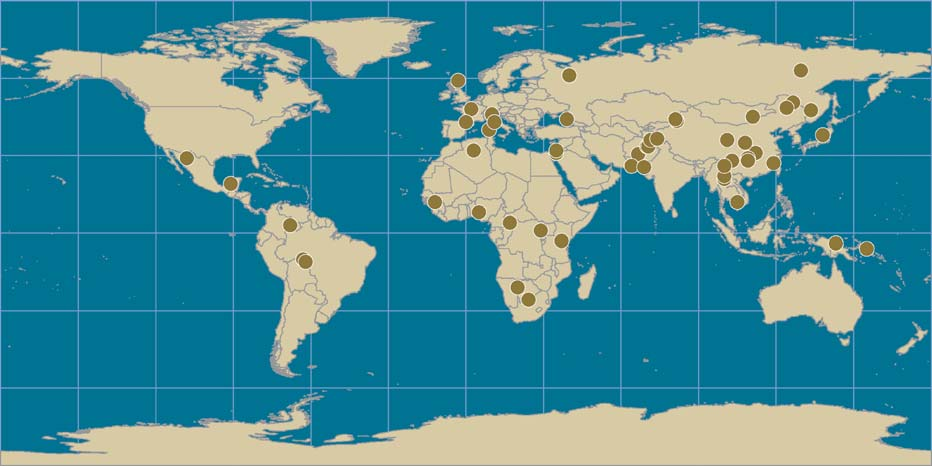
Worldwide Distribution of the 53 Populations of the CEPH Human Genome Diversity Cell Line Panel

**Figure 2:**
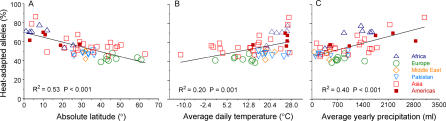
Heat Adaptation Is Strongly Associated with Absolute Latitude, Temperature, and Precipitation among the 53 Populations of the CEPH HGDP Cell Line Panel

Among the 53 populations represented in the Foundation Jean Dausset-Centre d'Etude du Polymorphisme Humain (CEPH) Human Genome Diversity Project (HGDP) (Figure 1), populations at low latitudes or in hot, wet climates, have a high prevalence of heat-adapted alleles, whereas populations at high latitudes or in cold, dry climates, have a low prevalence of heat-adapted alleles (Figure 2). For example, among populations within 10 degrees of the equator, 74%, on average, of the genetic variants are heat adapted. However, only 43% of the variants are heat adapted among populations within 10 degrees of the Arctic Circle. Across all populations, the association of heat adaptation with climate is strong, graded, and continuous.

**Figure 3:**
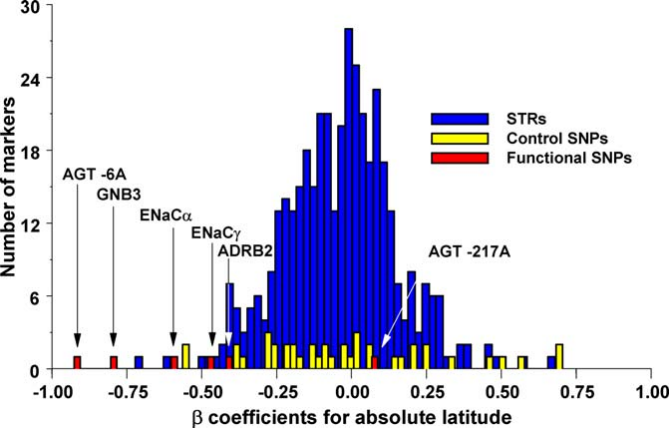
Histogram of β Coefficients for the Association of Absolute Latitude with Functional Genotypes, Neutral STR Markers, and Control SNPs for the 53 Populations of the CEPH HGDP Cell Line Panel

In order to assess the significance of the association between heat adaptation and latitude, we compared the worldwide distribution of the functional, heat-adapted alleles with the distribution of a set of 404 multiallelic short tandem repeat markers (STRs) and a set of 42 biallelic control SNPs. No STR or SNP allele had as strong an association with latitude as the functional alleles *AGT −6A* or *GNB3 825T* (Figure 3). Only one STR and two control SNPs were more strongly associated with latitude than *ENaCα −946G* and only seven STRs and eight control SNPs were more strongly associated with latitude than *ENaCγ −173G* or *ADRB2 47A/79c*.

If selection were responsible for the latitudinal cline in susceptibility, then the association should be strongest at the functional allele and decrease in a graded fashion at SNPs increasingly distant from the functional site. Therefore, we assessed the correlation between SNPs in and around each gene with latitude (Figure S1A–E). In *AGT, GNB3, ADRB2,* and *ENaCγ,* the strength of the correlation is greatest at the SNPs with the functional alleles and decreases in a graded fashion at SNPs increasingly distant from the SNP with functional alleles. In *ENaCα,* the strength of the correlation of *-946G* with latitude is second only to the closest neighboring SNP. Therefore, the latitudinal cline in heat adaptation is largely unmatched by latitudinal clines in STRs, control SNPs, or non-functional SNPs within the five genes.

Populations have experienced opposing selective gradients during the out-of-Africa expansion. Initially, heat-adapted African populations expanded northward, undergoing selection for cold adaptation. Subsequently, cold-adapted north Asian populations expanded southward into the Americas, undergoing selection for heat adaptation. Are latitudinal clines in heat adaptation similar in different regions of the world despite opposing directions of migration? The strength of the association of heat adaptation with latitude is similar among populations west of the Himalayas compared to populations in Asia (Table 1). Importantly, Native Americans, who are recently descended from cold-adapted north Asian populations [22,23], have a high percentage of heat-adapted alleles matching the level observed in African and Asian populations living at similar latitudes (see Figure 2, Table 1). This level of heat adaptation has occurred over the past 12,000 to 30,000 y and has been achieved despite the fixation of one SNP for the cold-adapted allele (*ENaCγ −173A*, Figure S2).

**Table 1:**
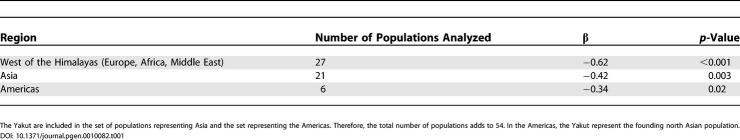
Association of Heat Adaptation with Absolute Latitude in Three Different World Regions among the 53 Populations of the CEPH HGDP Cell Line Panel

**Figure 4:**
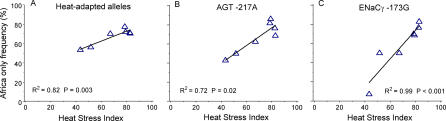
Heat Adaptation, *AGT G-217A,* and *ENaCγ* Are Strongly Associated with Heat Stress in Africa

**Figure 5:**
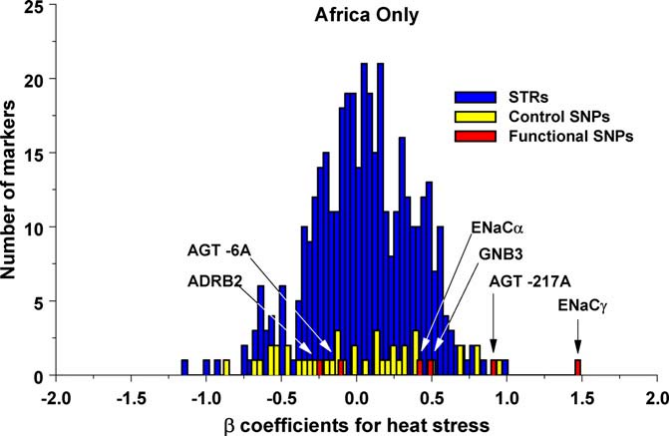
Histogram of β Coefficients for the Association of Heat Stress with Functional Genotypes, Neutral STR Markers, and Control SNPs for the Seven African populations of the CEPH HGDP Cell Line Panel

Although the association of heat adaptation with climate is consistent across regions, the relative contribution of each variant to heat adaptation varies. For example*, AGT −6A, GNB3 825T, ADRB2 47A/79C,* and *ENaCα −946G* contribute to worldwide variation in heat adaptation (Figure S2). In contrast, *ENaCγ −173G* makes a smaller contribution to worldwide variation and *AGT −217A* does not contribute. In Africa, however, *ENaCγ −173G* and *AGT −217A* are strongly associated with heat stress, which incorporates the combined effect of temperature and precipitation (*R^2^* = 0.99 *p* < 0.001, *R^2^* = 0.72 *p* = 0.02, respectively; Figure 4). The heat-adapted alleles are common among the rainforest-adapted Mbuti and Biaka pygmies and less common among groups living on the savanna such as the San. Furthermore, no STR or SNP allele had as strong an association with heat stress as *ENaCγ −173G* in Africa (Figure 5). Only three STRs and one control SNP allele were more strongly associated with heat stress than *AGT −217A* in Africa*.*

The ancestral African environment was characterized by a hot, wet climate and salt scarcity. Therefore, functional alleles that maximize adaptation to heat may be ancestral [24]. In the case of *AGT G-6A* and *ENaCγ,* the heat-adapted, functional alleles are ancestral (Table S1). For *AGT G-217A, GNB3, ADRB2,* and *ENaCα,* however, the heat-adapted alleles are derived. Therefore, although a portion of hypertension susceptibility is ancestral, salt avidity and cardiovascular reactivity were likely magnified during early human evolution prior to the out-of-Africa expansion of anatomically modern humans. Increasing aridification in East Africa and subsequent adaptation to savannah may have driven the magnification of salt avidity and cardiovascular reactivity in early *Homo*.

### *GNB3 C825T* and Latitude Are Strongly Associated with Worldwide Variation in Blood Pressure

If selection shaped our susceptibility to hypertension through ecological variables that correlate with latitude, then latitude and the associated functional alleles should have a measurable effect on worldwide variation in blood pressure. In order to test this hypothesis, we investigated the extent to which latitude and hypertension susceptibility contribute to worldwide blood pressure variation using data from INTERSALT, an epidemiologic study of blood pressure in 52 populations around the world. We estimated the population frequency of *GNB3 825T* for 35 INTERSALT populations using data from HGDP and the literature (Table S2).

**Figure 6:**
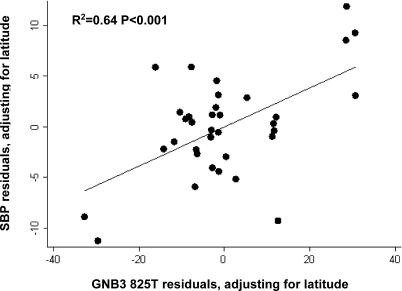
Residual Plot Demonstrating the Association of *GNB3 825T* with Systolic Blood Pressure after Adjustment for Latitude among 35 INTERSALT Populations with Matching Genotype Data

Among the 35 INTERSALT populations with allele frequency data, latitude explained 47% of the worldwide variation in blood pressure (Table S3). With an increase of one degree of latitude north or south, population-average systolic blood pressure increased by 0.3 mmHg. When *GNB3 825T* was included in the analysis, we were able to explain 64% of worldwide variation in blood pressure (Table 2, Figure 6). With each 1% increase in *GNB3 825T* allele frequency, blood pressure increased 0.19 mmHg. When body mass index (BMI) was added to the model, 74% of the variation in mean systolic blood pressure was explained. The addition of sodium excretion, BMI, or alcohol intake did not diminish the effect of latitude and *GNB3 825T* on blood pressure variation (data not shown).

**Table 2:**
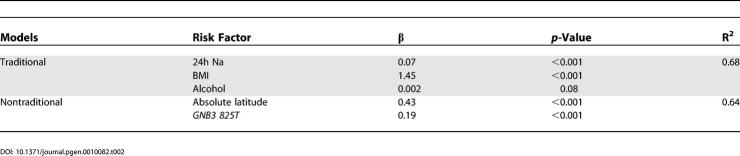
Multivariate Models Describing the Association of Traditional and Nontraditional Risk Factors with Systolic Blood Pressure (mm Hg) among 35 INTERSALT Populations with Allele Frequency Data

The strong association of latitude and *GNB3 825T* frequency with worldwide blood pressure variation is partly due to the use of population-level data in this analysis. Latitude represents a number of ecological factors that affect blood pressure, such as temperature and humidity. Likewise, *GNB3 825T* represents a number of functional alleles that influence hypertension susceptibility. For example, those populations in HGDP that have a high prevalence of the *GNB3 825T* allele also have a high prevalence of heat-adapted alleles at other SNPs (correlation of *GNB3 825T* with heat adaptation = 0.82). At present we are unable to assess the impact of other alleles on blood pressure in INTERSALT, because frequencies for other functional alleles were available for only a few INTERSALT populations. It is highly likely, however, that these and other unmeasured hypertension susceptibility alleles that correlate with latitude contribute to the predictive strength of *GNB3 825T*.

**Figure 7:**
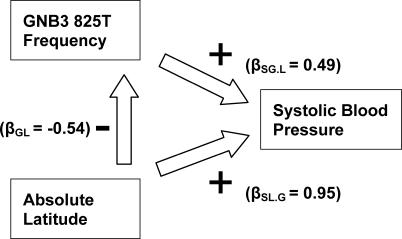
Epidemiologic Model Relating Absolute Latitude and Population-Average *GNB3 825T* Frequency with Each Other and with Population-Average Systolic Blood Pressure among 35 INTERSALT Populations with Matching Genotype Data The β coefficients represent those in the models listed in Table 3.

*GNB3 825T* frequency is not associated with blood pressure until after adjustment for latitude (Table 3, Figure 7). Likewise, the association of latitude with blood pressure increases when *GNB3 825T* is included in the model. When the association between two variables is increased by a third, the third variable is said to negatively confound the relationship between the other two. Therefore, *GNB3 825T* and latitude negatively confound each other in their relationship with blood pressure. Negative confounding occurs because at low latitudes, blood pressure tends to be low and *GNB3 825T* frequency tends to be high, whereas at high latitudes blood pressure tends to be high and *GNB3 825T* frequency tends to be low. Therefore, in unadjusted analyses, the effect of *GNB3 825T* on blood pressure is counterbalanced by the effect of latitude on blood pressure. When the counterbalancing effect of latitude is removed through adjustment, however, *GNB3 825T* is strongly associated with blood pressure. Likewise, after adjustment for *GNB3 825T,* the association of latitude with blood pressure is strengthened as well.

**Table 3:**
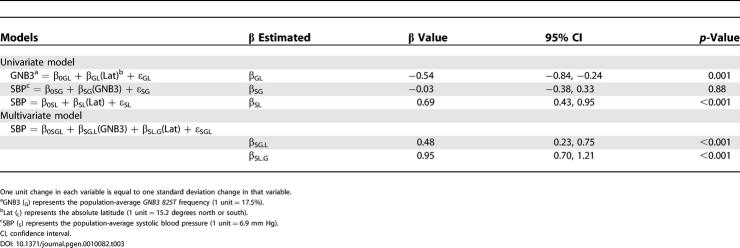
Epidemiologic Models Describing the Relationships among Absolute Latitude, *GNB3 825T,* and Systolic Blood Pressure among 35 INTERSALT Populations with Genotype Data

What is the source of this negative confounding? As discussed above, other functional alleles may contribute to the effect of *GNB3 825T* on blood pressure. These alleles would be positive rather than negative confounders, however, decreasing rather than increasing the association of *GNB3 825T* with blood pressure. The observed negative confounding is more likely the result of natural selection. As populations migrated to higher latitudes during the out-of-Africa expansion, population-average blood pressure increased. Natural selection favored those not in the upper range of blood pressure and *GNB3 825T* frequency decreased. The population-level effect is that average *GNB3 825T* frequency is negatively correlated with latitude. In addition, depending on time elapsed and the efficiency of natural selection, blood pressure will tend not to vary by latitude or genotype, because across populations, the effect of *GNB3 825T* balances the effect of latitude. Therefore, the observed interdependence of latitude, *GNB3 825T,* and blood pressure is likely due to natural selection.

## Discussion

We have shown that populations differ in susceptibility to hypertension and provide compelling evidence that this differential susceptibility is due to differential exposure to selection pressure since the out-of-Africa expansion. The most important selection pressure was climate, which produced a latitudinal cline in susceptibility. Consistent with this hypothesis, latitude and hypertension susceptibility as determined by the presence of the *GNB3 825T* allele explains a large portion of worldwide variation in blood pressure.

A growing body of evidence suggests that selection has shaped genetic susceptibility to several common diseases [3–7,20,21,25]. As reviewed by Di Rienzo and Hudson [24], many alleles that increase susceptibility to hypertension, coronary artery disease, and type 2 diabetes are ancestral, rather than derived. This ancestral susceptibility is consistent with the notion that alleles that were adaptive in our ancestral environment are maladaptive in the current one. As shown here, however, many alleles that increase susceptibility to hypertension are not ancestral, suggesting that hypertension susceptibility was magnified after the divergence of the line leading to *Homo* approximately 6 million y ago. Consistent with this idea, many of the features that differentiate humans from non-human primates, such as a tall linear body frame, hairlessness, and an enhanced capacity to sweat, facilitate heat dissipation [26,27]. Increasing aridification in East Africa and subsequent adaptation to savannah may have driven the evolution of these heat-adapted features, as well as the magnification of salt avidity and cardiovascular reactivity in early *Homo*.

Originating in Africa, our species has since expanded out of Africa to inhabit the rest of the world. This exposure to widely divergent ecological conditions may have contributed to human diversification. Recently, Ruiz-Pesini et al. found evidence of selection by latitude in human mitochondrial genes [28]. This variation may influence the efficiency of oxidative phosphorylation and, therefore, cellular heat production, an important adaptation to external temperature. Comparative physiologists have long recognized the importance of selection by latitude. In 1847, Bergmann [29] described a latitudinal cline in body size and shape that is common for species with a wide geographic range [30]. In fact, the association is stronger for humans than for many other species [31]. Allen described a similar latitudinal cline in limb length such that as latitude increases, limb length decreases [32]. This association is also found in humans [26,31,33]. Both ecological rules serve to facilitate heat dissipation at the equator by maximizing body surface area to volume ratio and to conserve heat toward the poles by minimizing this ratio. The effectiveness of increased surface area to dissipate heat is further maximized through evaporative heat loss, made possible by increased salt avidity and cardiovascular reactivity. As populations expanded into colder climates, the primary thermodynamic requirement shifted from heat dissipation to heat conservation, and selection for salt avidity and cardiovascular reactivity lessened. Therefore, differential susceptibility to hypertension may be a consequence of ecological selection during the out-of-Africa expansion. Consistent with this notion, Nakajima et al. found evidence of positive selection among populations outside of Africa near one of the SNPs examined in this work, *AGT G-6A* [20]. Similarly, Thompson et al. found evidence of positive selection near another SNP associated with increased salt avidity and hypertension, CYP3A5*1/*3 [21]. We further demonstrate that latitudinal variation in hypertension susceptibility is not limited to salt avidity and is widespread, involving a functional variant in all five genes examined.

The rapidity of physical adaptation to climate is illustrated by the changes in body shape that occurred in Europe over the past 30,000 y. Prior to the Last Glacial Maximum, people with body proportions approximating those of savannah-adapted equatorial groups expanded into Europe. Within 20,000 y, these populations had developed high-latitude body proportions characterized, in part, by a smaller limb length to trunk ratio [26,34]. Indeed, the evolution of clinal variation in body size and shape has occurred throughout Eurasia and, to a limited degree, among native populations in the Americas [26,31]. Similarly, Native American populations demonstrate a level of salt avidity and cardiovascular reactivity equal to populations at similar latitudes in Africa, despite their recent descent from cold-adapted north Asian populations. This level of heat adaptation has occurred over the course of less than 20,000 y, attesting to the strength of selection by latitude.

Latitude and hypertension susceptibility, as measured by *GNB3 825T* frequency, explain a large portion of worldwide variation in blood pressure. Importantly, the effect of *GNB3 825T* is not appreciated until latitude is included in the model. This negative confounding is likely a result of natural selection. As populations expanded north out of Africa, salt avidity and cardiovascular contractility decreased as latitude increased, thereby limiting the upward shift in population-average blood pressure due to latitude. In other words, natural selection acted as a homeostatic mechanism preserving blood pressure by matching genes to environment. This negative confounding by natural selection, as well as the strength of the collective effect of latitude and *GNB3 825T* on worldwide variation in blood pressure, are strong evidence that latitude influenced worldwide variation in hypertension susceptibility through selection.

What is the source of the current epidemic of hypertension among industrialized populations? As shown in migration studies, the shift in the population average blood pressure occurs as people migrate into hypertensinogenic environments [35,36]. Therefore, the upward shift in blood pressure distribution among industrialized populations is more likely due to a greater burden of exposures such as increased salt intake and obesity than increasing hypertension susceptibility. However, we have shown that populations differ in susceptibility to these exposures. In addition, our analysis suggests that the migration of heat-adapted people to colder climates may contribute to the upward shift in population distribution of blood pressure, especially among members of the African Diaspora. Finally, the evolutionary history of disease susceptibility has important implications for association studies. As described by Di Rienzo and Hudson [24], protective alleles may be easier to identify than older hypertension susceptibility alleles because protective alleles have risen to high frequency more recently and, therefore, are more tightly linked to nearby markers.

In summary, we have shown that differential susceptibility to hypertension is due to differential exposure to selection pressure since the out-of-Africa expansion. The current epidemic of hypertension is due to the new exposures of the modern period, such as higher salt intake, interacting with ancestral susceptibility. However, populations differ in susceptibility to these new exposures such that those from hot environments are more susceptible to hypertension than populations from cold environments. This differential susceptibility is likely due to our history of adaptation to climate.

## Materials and Methods

### Human DNA.

DNA was obtained from the CEPH HGDP panel representing 1,064 people from 53 populations around the world (see Figure 1). Populations that had undergone recent migration were excluded. Participants were descended from several generations of ancestors who belonged to the same population. The Han samples were divided into two groups, Han from North China and Han from Central China.

### Genes and their functional alleles.

We chose seven SNPs with functional alleles in five genes based upon three criteria. First, the gene must influence salt and water avidity or cardiovascular reactivity. Second, variation at the SNP must affect gene expression or function in experimental studies. Third, the minor allele frequency must be greater than 5% worldwide. The selected genes include *AGT, GNB3, ADRB2,* and the epithelial sodium channel components *ENaCα* and *ENaCγ* (see Accession Numbers and Table S1). Below is a description of these genes and the evidence for their inclusion in this work.

Angiotensinogen is the precursor of angiotensin II, which increases arterial tone and salt avidity. The substitution of adenine for guanine in the promoter *(G-6A)* increases the gene's transcription rate and is associated with increased angiotensinogen levels and higher blood pressure [37,38]. The SNP *M235T* was the first molecular variant in *AGT* found to have an association with hypertension. However, there are no published experimental data suggesting that the two alleles have differential effects on gene expression or function. Rather, the association of *235T* with hypertension is likely due to linkage with *-6A*. Therefore, we have not included *235T* as one of our heat-adapted alleles. The *AGT* promoter has a second SNP with functional alleles, *AGT G-217A,* which is associated with hypertension among African Americans [39,40]. Substitution of adenine for guanine in transfection studies increases gene transcription and promoter activity induced by glucocorticoids and the *C/ERB* family of transcription factors [39,40].

Salt avidity is a function of epithelial sodium channel activity, which is the rate-limiting step in renal sodium reabsorption. Mutations in *ENaC'*s three subunits *α, β,* and *γ* (also called *SCNN1α, β,* or *γ*) are responsible for monogenic forms of hypertension. More common *ENaC* polymorphisms may be responsible for population differences in salt avidity. Inwai et al. found that the substitution of guanine for adenine in the promoter of the *ENaCα* subunit *(A-946G*, also called *A2139G)* increases the promoter activity in a cell-based transfection assay and is associated with hypertension [41]. The same group found a functional polymorphism in the promoter of the *ENaCγ* subunit [42]. The substitution of guanine for adenine at position *-173* (*A-173G*) increased promoter activity in a similar transfection assay and was associated with higher blood pressure [42].

G protein beta3 subunit is one of five distinct β subunits that, in combination with α and γ subunits, form signal-transducing G proteins. G proteins couple cell surface receptors to effectors that generate intracellular signals, thereby regulating many biological functions, including blood pressure [43]. A *GNB3* subunit containing thymine *(825T)* rather than cytosine *(825C)* at cDNA position *825* is associated with a truncated splice variant [44]. Functional analyses of the splice variant in model systems demonstrate enhanced signal transduction and Gα activation [45]. In human studies, vasoactive agents such as endothelin-1, angiotensin II, and noradrenaline produce greater vasoconstriction in people with the *825T* allele [44]. In addition, *825T* is associated with hypertension in many, but not all, populations ([46] provides a summary; please see Table S1 for other references).

The β2-adrenergic receptor is responsible for catecholamine-mediated vasodilatation. In the setting of volume depletion, excess vasodilatation in response to increased sympathetic tone would compromise blood pressure. Therefore, *ADRB2* variation that decreases this vasodilatory response would be advantageous in hot environments. Two base pair substitutions, *A47G* and *C79G,* result in two functional amino acid substitutions, Arg16Gly and Gln27Glu respectively. Both substitutions affect receptor desensitization with prolonged agonist activation in vitro [47,48]. In populations, however, only three of the four possible combinations occur, *47A/79C, 47G/79C,* and *47G/79G* [49]. Therefore, Dishy et al. investigated the affect of each combination in human subjects and found that *47A/79C* was associated with increased desensitization to the vasodilatory affect of isoproterenol [49]. Similarly, we have assigned one of three possible *ADRB2 A47G C79G* haplotypes to each HGDP participant. We were able to do so unambiguously because the haplotype *47A/79G* does not occur.

### SNP genotyping.

We used a previously described 5′ nuclease allele discrimination assay (ABI TaqMan; Applied Biosystems, Foster City, California, United States) for all SNPs except *M235T* (rs699). Genotyping for *M235T* was performed using template directed extension coupled with detection by fluorescence polarization. Oligonucleotide sequences for all genotyping assays are available upon request.

### The HGDP-CEPH analysis.

To test the hypothesis that populations differ in heat adaptation, we estimated individual level and population level of heat adaptation. In the case of *AGT, GNB3, ENaCα,* and *ENaCγ,* the heat-adapted variants are the alleles that increase sodium avidity or vascular tone. In the case of *ADRB2,* the heat-adapted variant is the haplotype *27A/47C,* which increases receptor desensitization. Individuals were scored for heat adaptation on the basis of the number of heat-adapted variants present divided by the number of possible heat-adapted variants. The total number of possible heat-adapted variants was the number of genotyped SNPs in an individual times two. Population-level susceptibility is defined as the average susceptibility of its members.

To test the hypothesis that differential heat adaptation is due to selection during the out-of-Africa expansion, we used linear regression to estimate the association of each functional variant with latitude, temperature, and precipitation. Heat-adapted alleles were hypothesized to be more common at latitudes near the equator and in areas with higher temperature and precipitation. In addition, we combined temperature and precipitation into a single term labeled heat stress. First, both variables were log-transformed to create normal distributions (temperature in degrees Kelvin). Second, we generated units equal to one standard deviation to make a unit increase in each variable comparable to the other. Third, we multiplied the variables together to create the summary variable. Finally, the summary variable heat stress was normalized by dividing by the highest level of heat stress in this dataset to produce a percentage.

In order to ascertain whether any observed associations were due to genetic drift or nonspecific selection, we compared variation in functional alleles to worldwide variation in 404 STRs, to variation in 42 control SNPs, and to variation in other nonfunctional SNPs in each gene. First, we used STRs previously genotyped by the Mammalian Genotyping Service using the Marshfield Screening Set #10 (http://research.marshfieldclinic.org/genetics/sets/combo.html). Results from this dataset have been previously reported [50] and are available online. Because STRs are not biallelic, we determined the major allele at each STR and then the frequency of that allele in each population. Second, we genotyped a set of control SNPs that were approximately frequency matched to the SNPs with functional alleles. From Applied Biosystems Assays on Demand, we chose 42 control SNPs, approximately one per chromosome arm, with a minor allele frequency near 40%. Assay IDs are available upon request. Third, we assessed the correlation between latitude and allele frequencies at polymorphic sites in and around each gene.

### The INTERSALT data.

All phenotypic data were taken from data previously published by the INTERSALT investigators [51]. INTERSALT was an international study of blood pressure variation among 52 populations performed in the late 1980′s. Approximately 200 men and women aged 20–59 y were recruited from each population, giving a total of 10,079 people in all. Phenotypic data included population mean systolic blood pressure, diastolic blood pressure, and BMI (weight in kilograms divided by height in meters squared). Blood pressure was measured twice with a random zero sphygmomanometer using a standard protocol by trained personnel. Exposure data included population mean 24-h urine sodium excretion and alcohol intake. Height and weight were also measured twice. Participants provided a 24-h urine collection for electrolyte analysis. Using 13 sources, including HGDP and the literature, we were able to estimate *GNB3 825T* allele frequency for 35 INTERSALT populations (references in Table S2). Of the 52 INTERSALT populations, the investigation by Siffert et al. of the worldwide distribution of *GNB3* 825T contributed to the allele frequency estimation for 25 populations [52]. The HGDP contributed information for seven populations, and other population-based sources contributed information for 13 populations. More than one source contributed information for 12 INTERSALT populations. In these cases, the allele frequency estimation for the INTERSALT populations was based on a weighted average of the allele frequency estimates. We were not able to estimate the frequency of other functional alleles in a sufficient number of INTERSALT populations.

### The INTERSALT analysis.

We used linear regression to describe the association of latitude and *GNB3 825T* with blood pressure, with and without adjustment for BMI, sodium excretion, and alcohol intake. The study populations were recruited by age and gender strata. Therefore, we did not adjust for these variables.

In our analysis of negative confounding, we used linear regression to describe the association of *GNB3 825T* with blood pressure, with and without adjustment for latitude, latitude with blood pressure, with and without adjustment for *GNB3 825T,* and latitude with *GNB3 825T.* In order to generate β coefficients that are comparable, we rescaled the variables such that a one unit change in a variable was equal to a one standard deviation change in the variable.

## Supporting Information

Figure S1 Association between Allele Frequencies for *AGT, GNB3, ADRB2, ENaCα,* and *ENaCγ* and Absolute Latitude among the 53 Populations of the CEPH HGDP Cell Line Panel (A) *AGT,* (B), *GNB3,* (C) *ADRB2,* (D) *ENaCα,* and (E) *ENaCγ.* The tick bars in the gene diagram represent the region from the first to the last exon. The bar under the diagram represents physical distance corresponding to 5 kb, except for *ADRB2* where the bar represents 0.5kb. The diamonds represent SNPs. The corresponding histogram bar is indicated by the connecting line.
(72 KB PDF)

Figure S2 The Association of Absolute Latitude with the Functional Genotypes in Five Genes Involved in Blood Pressure Regulation among the 53 Populations of the CEPH HGDP Cell Line Panel (55 KB PDF)

Table S1 The Selected Genes and Their Functional SNPs (34 KB DOC)

Table S2 Population Averages of Phenotype and Allele Frequency Data among the 35 INTERSALT Populations with Estimated *GNB3 825T* Allele Frequency (91 KB DOC)

Table S3 Univariate Models Describing the Association of Traditional and Nontraditional Risk Factors with Population-Average Systolic Blood Pressure (mm Hg) among 35 INTERSALT Populations with Genotype Data (30 KB DOC)

### Accession Numbers

The National Center for Biotechnology Information (NCBI) (http://www.ncbi.nlm.nih.gov) accession numbers for the genes discussed in this paper are *ADRB2* (GeneID 154), *AGT* (GeneID 183), *GNB3* (GeneID 2784), *ENaCα* (GeneID 6337), and *ENaCγ* (GeneID 6338).

## Abbreviations

BMI: body mass index
CEPH: Foundation Jean Dausset-Centre d'Etude du Polymorphisme Humain
HGDP: CEPH Human Genome Diversity Program
SNP: single nucleotide polymorphism
STRs: short tandem repeat markers
