# Supplementary figures and images for: Differential Susceptibility to Hypertension Is Due to Selection during the Out-of-Africa Expansion
Source: PLoS Genet. 2005 Dec 30;1(6):e82. doi: 10.1371/journal.pgen.0010082 (PMC1342636; doi:10.1371/journal.pgen.0010082)

**A****AGT**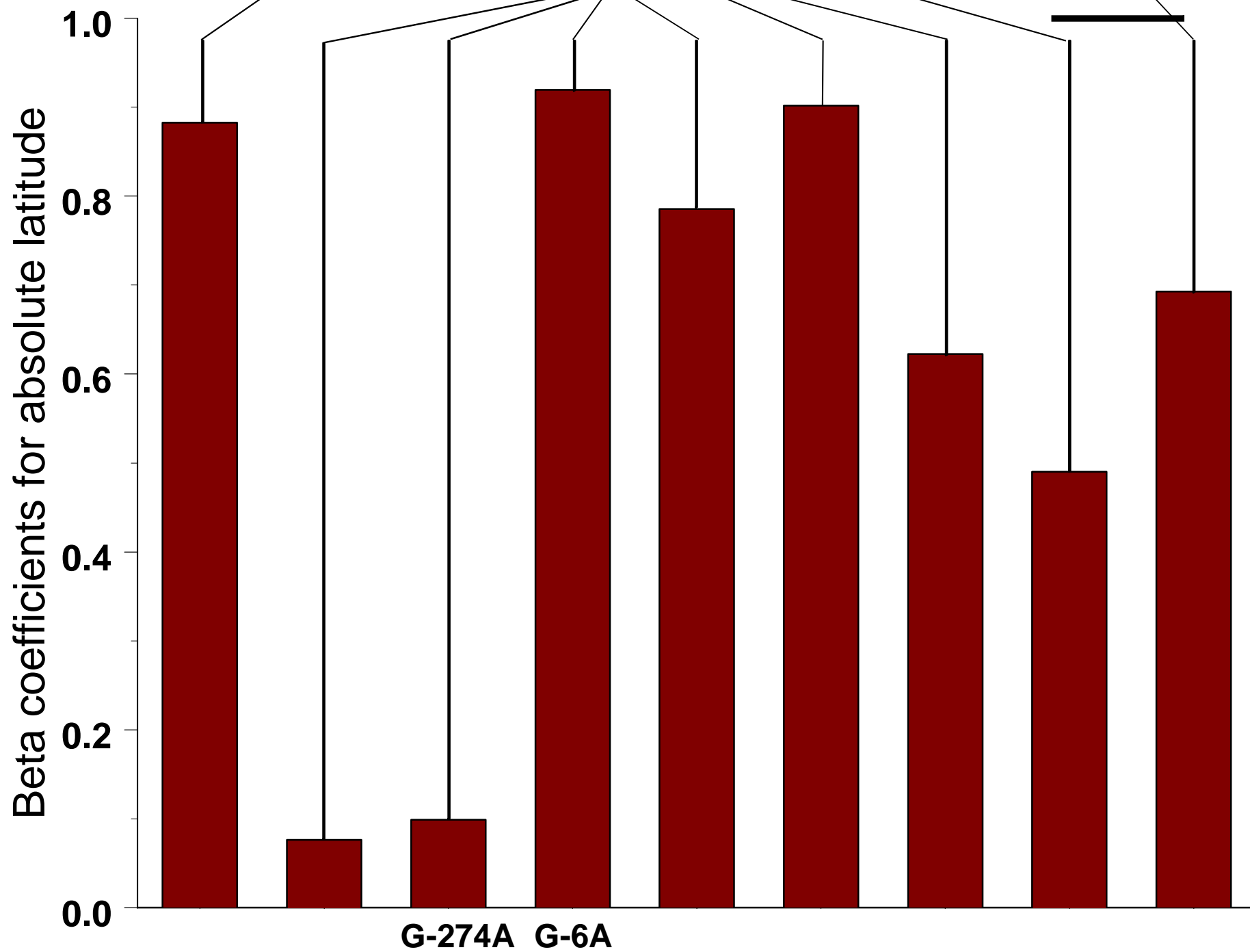

**B****GNB3**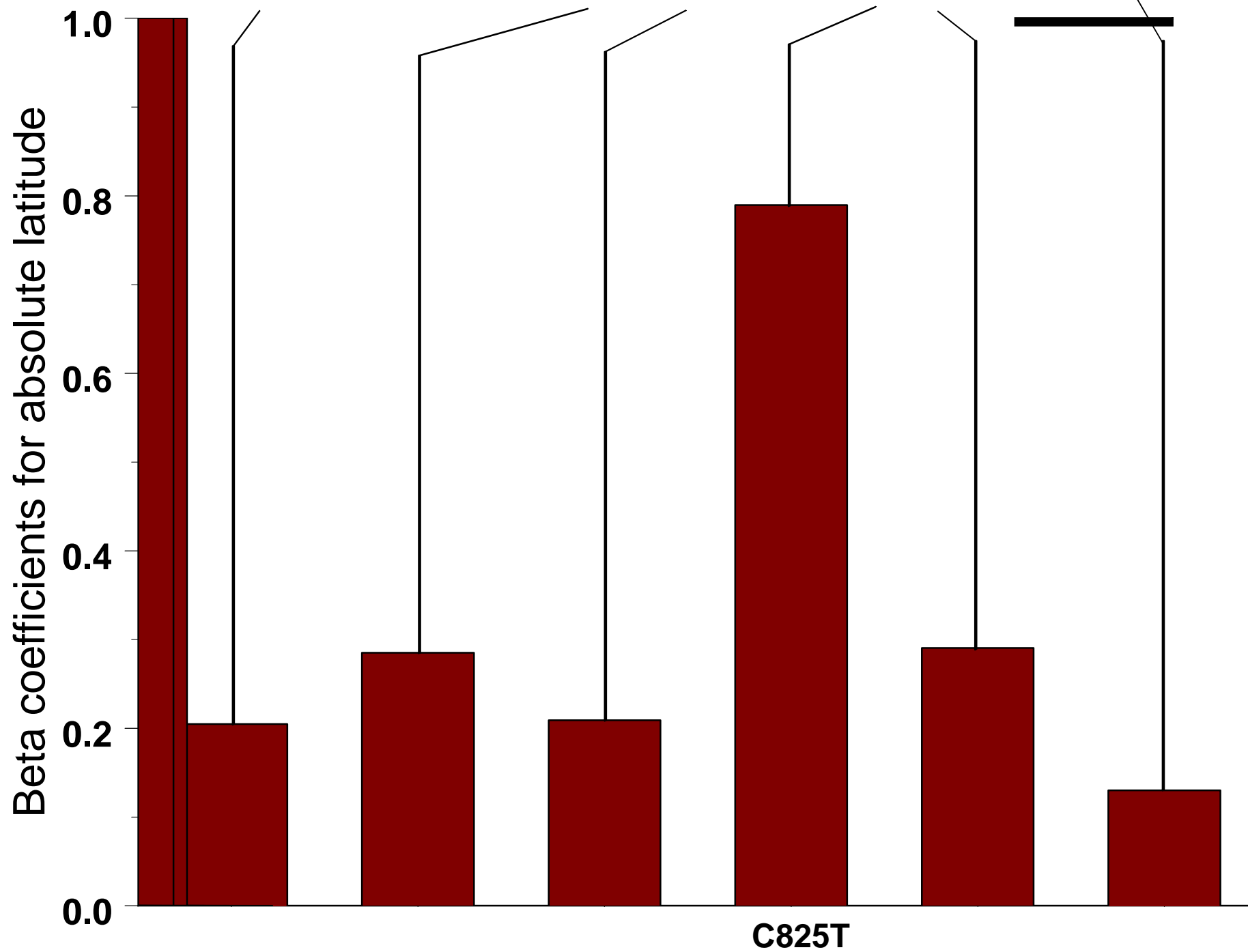

**C****ADRB2**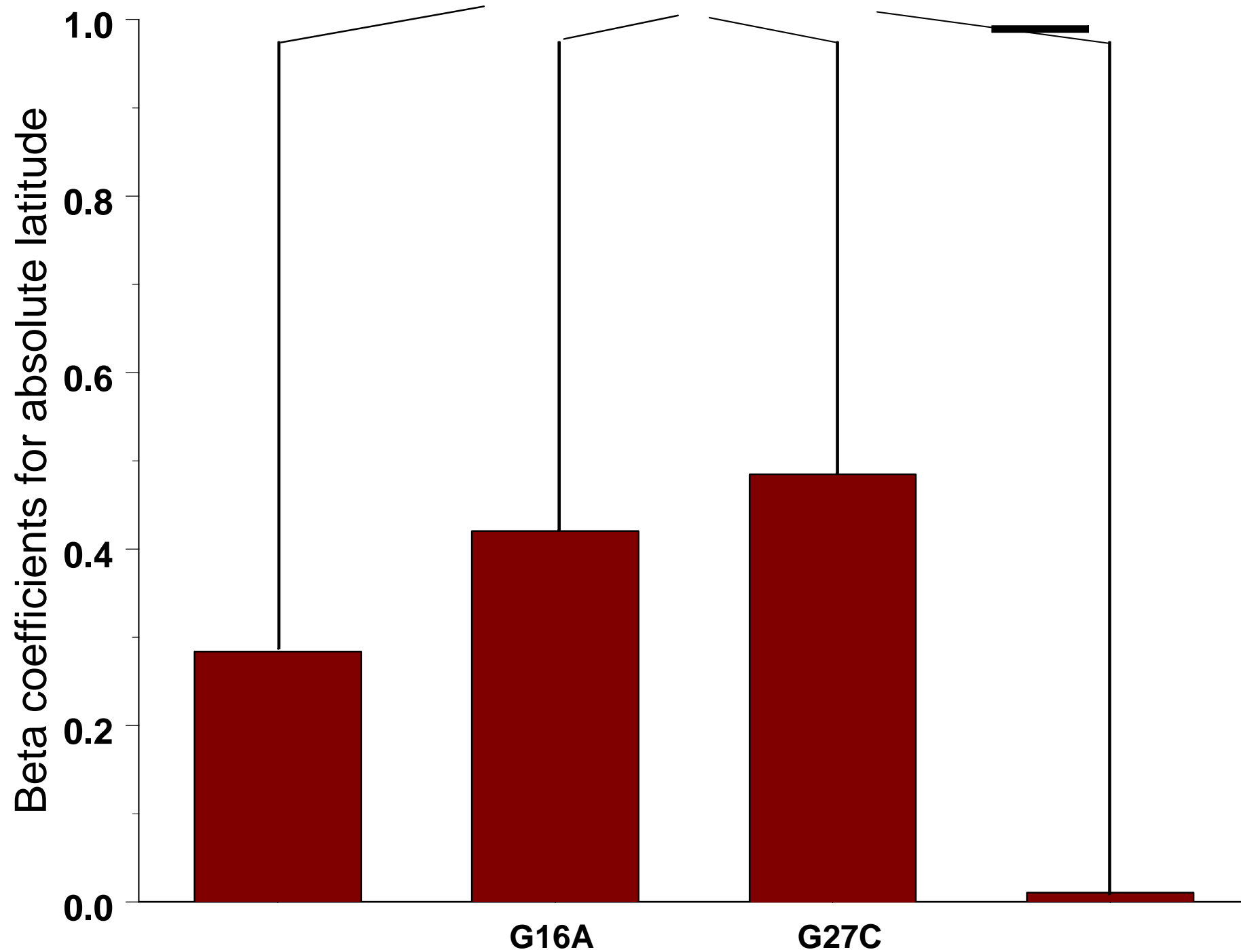

**D****ENaC $\alpha$** 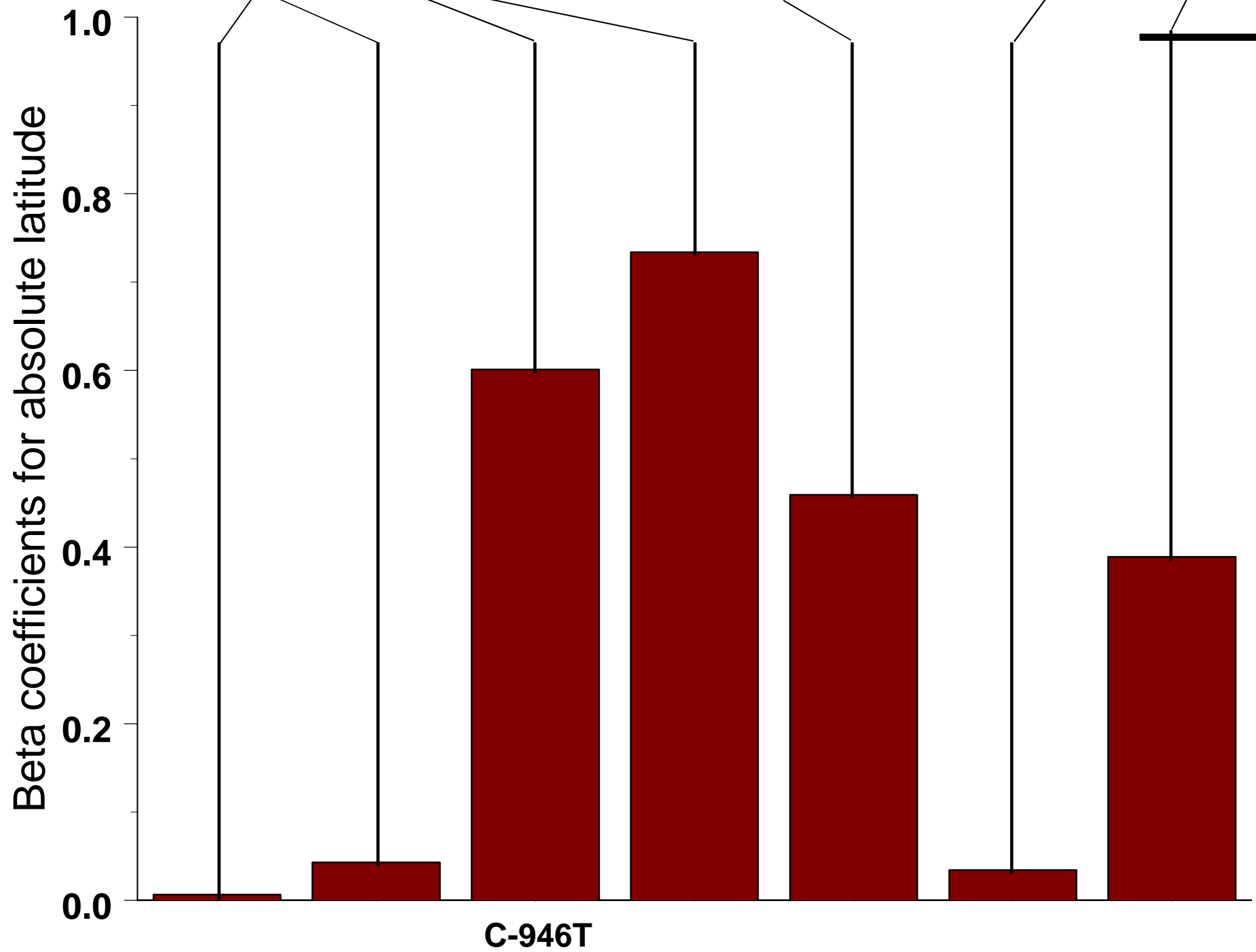

E

ENaC $\gamma$

Beta coefficients for absolute latitude

1.0  
0.8  
0.6  
0.4  
0.2  
0.0

A-173G

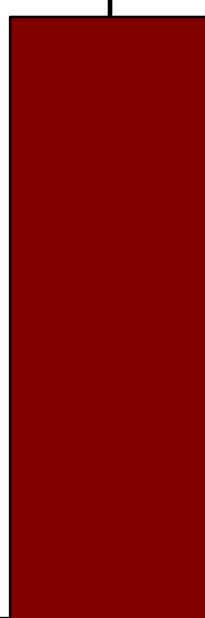

Supplement: Figure S1 — (A) AGT, (B), GNB3, (C) ADRB2, (D) ENaCα, and (E) ENaCγ. The tick bars in the gene diagram represent the region from the first to the last exon. The bar under the diagram represents physical distance corresponding to 5 kb, except for ADRB2 where the bar represents 0.5kb. The diamonds represent SNPs. The corresponding histogram bar is indicated by the connecting line. (72 KB PDF) [file pgen.0010082.sg001.pdf]

**A**

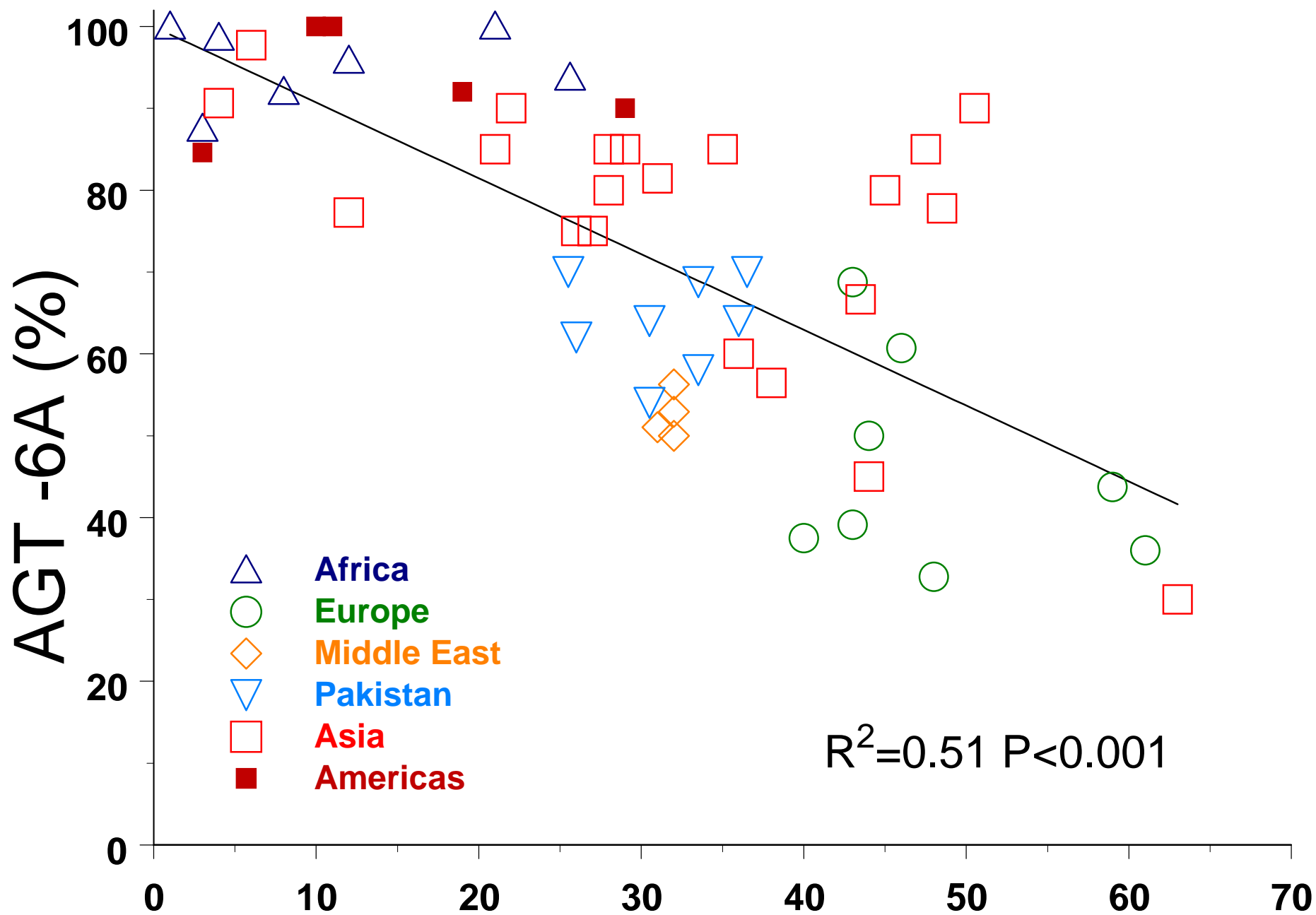

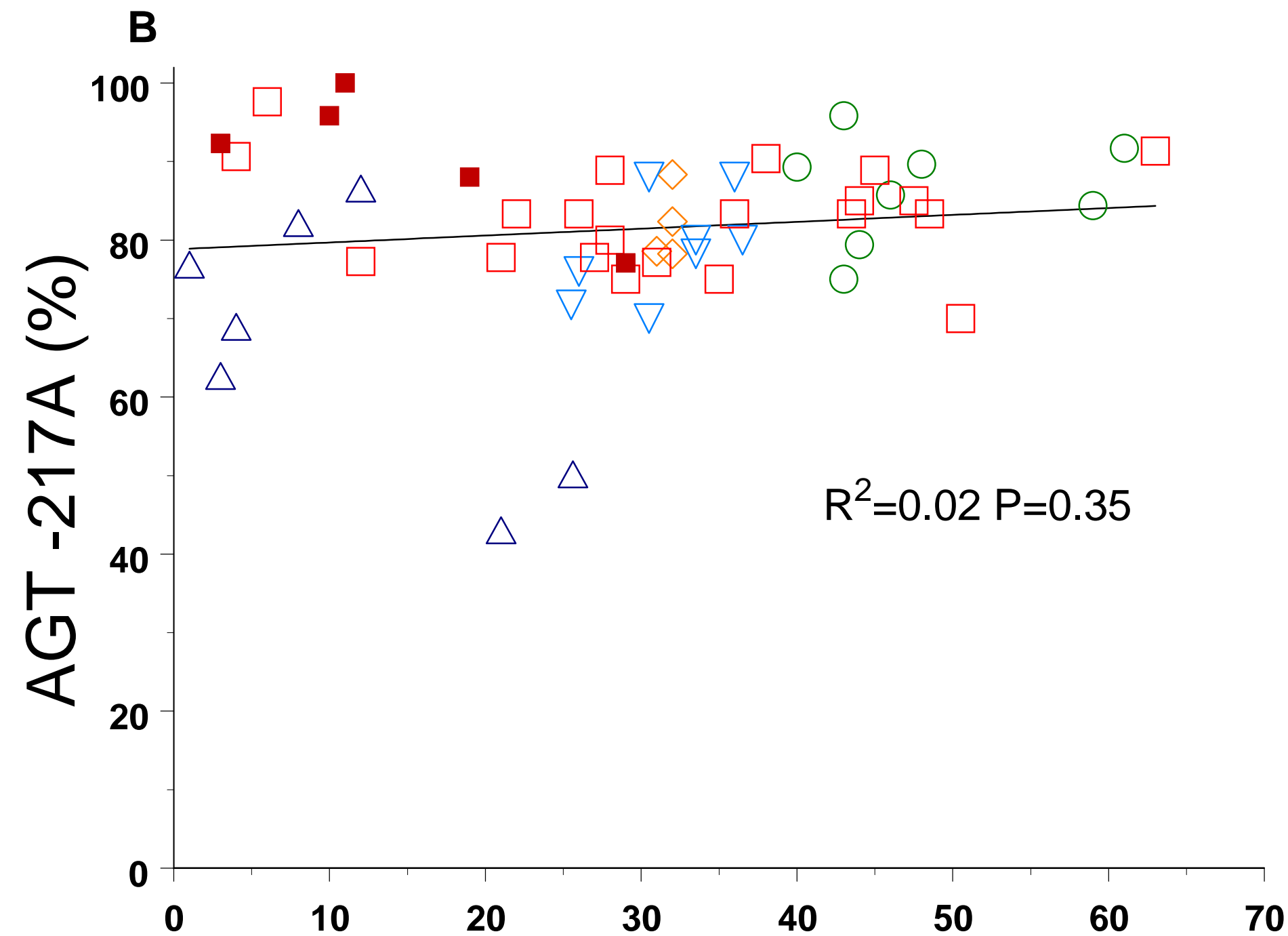

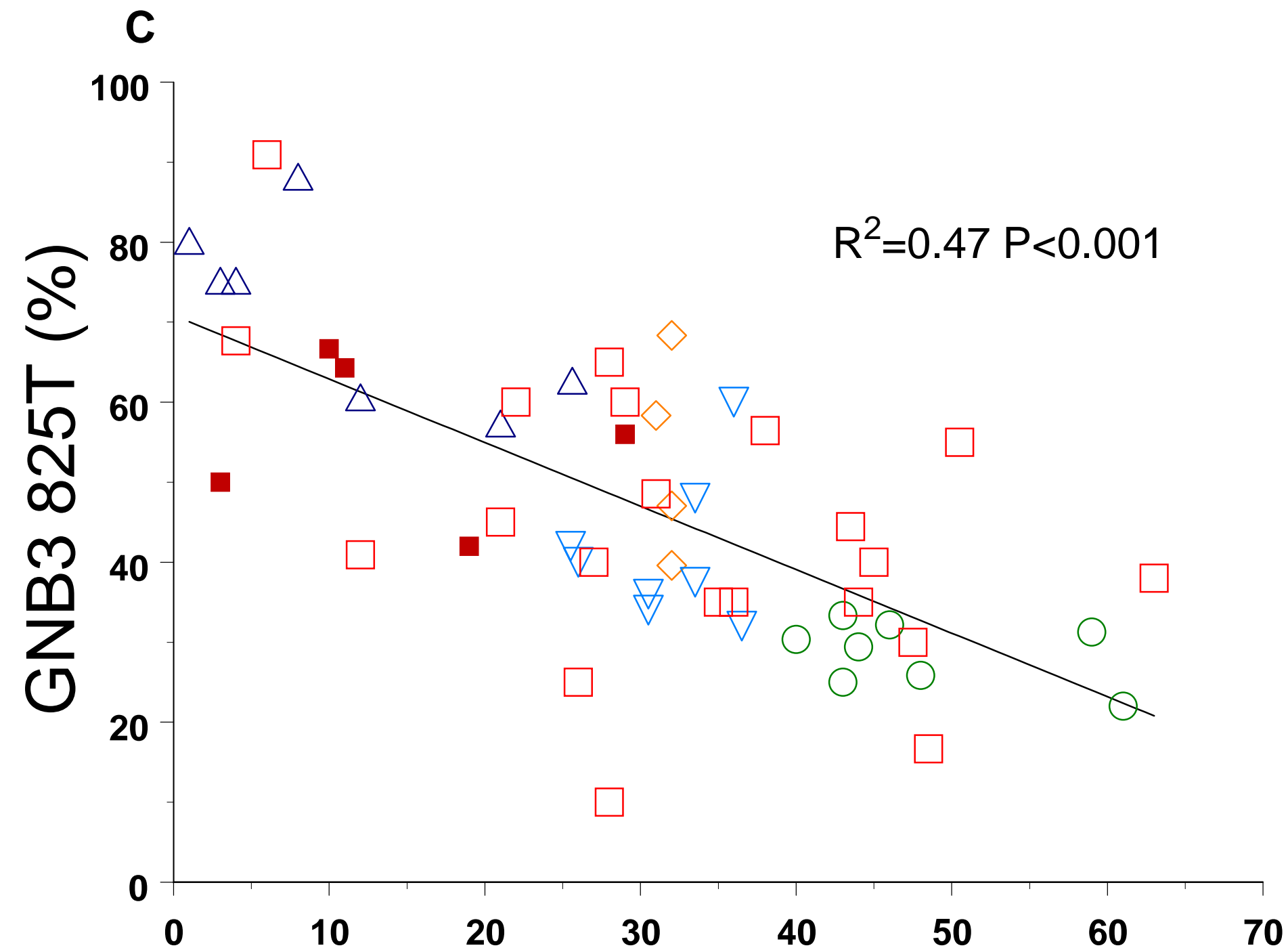

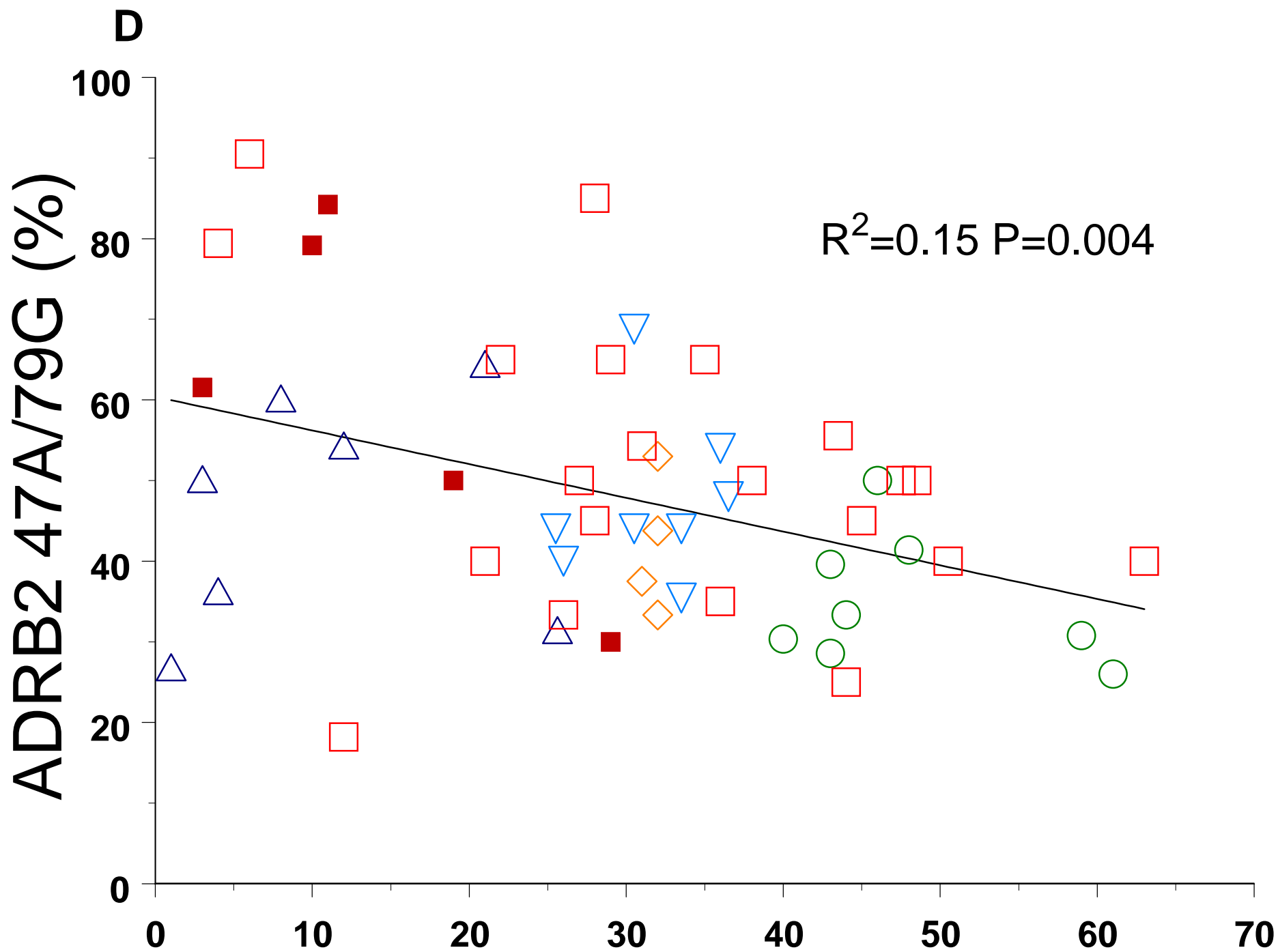

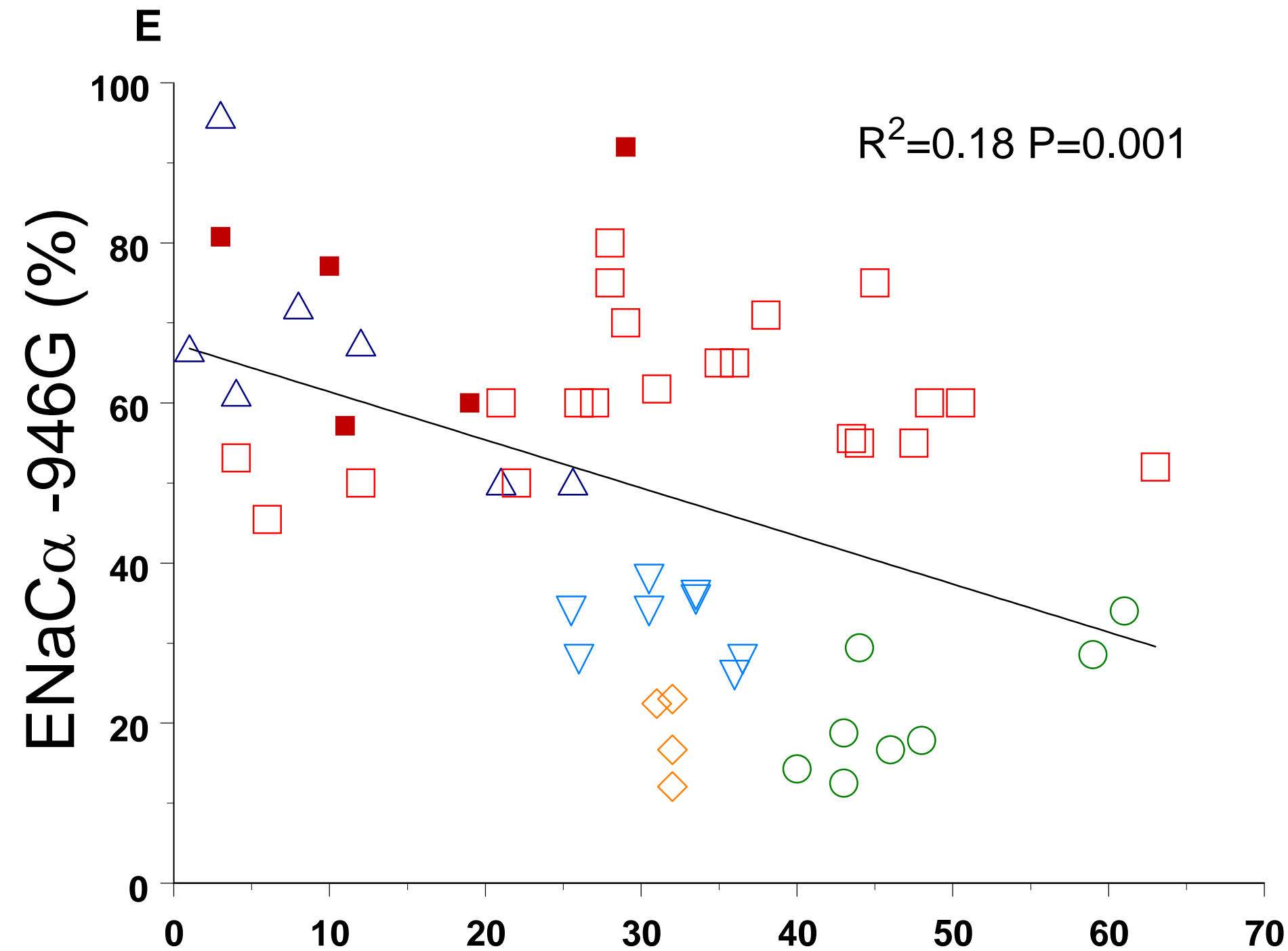

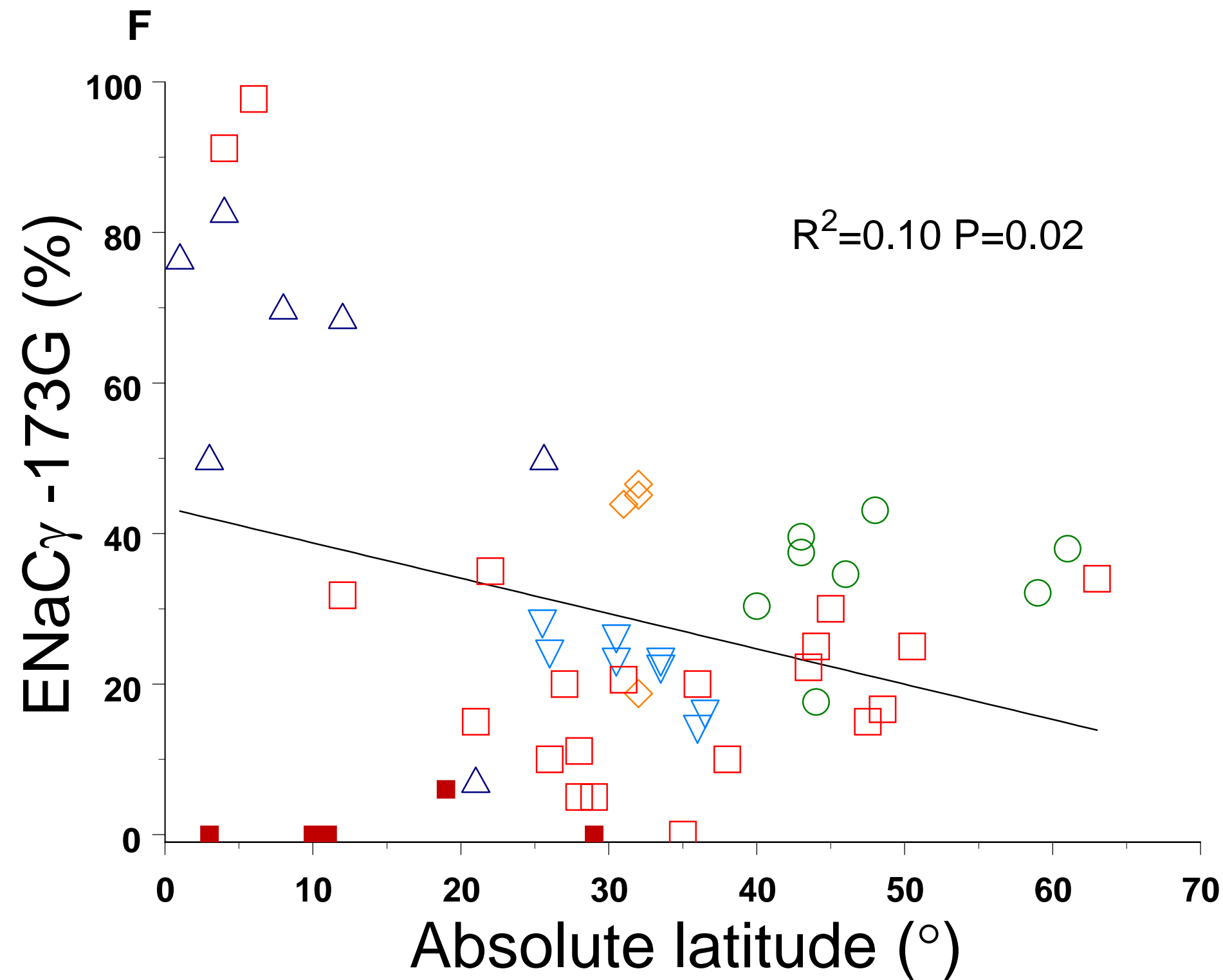

Supplement: Figure S2 — (55 KB PDF) [file pgen.0010082.sg002.pdf]
