# Supplementary material for: Differential Susceptibility to Hypertension Is Due to Selection during the Out-of-Africa Expansion
Source: PLoS Genet. 2005 Dec 30;1(6):e82. doi: 10.1371/journal.pgen.0010082 (PMC1342636; doi:10.1371/journal.pgen.0010082)
Supplement: Table S1 — (34 KB DOC) [file pgen.0010082.st001.doc]

| Table S1: The selected genes and their functional SNPs. | | | | | | |
| --- | --- | --- | --- | --- | --- | --- |
| Gene (common symbol) | NCBI GeneID | SNP ID | SNP position | Alleles | Ancestral alleles | Heat adapted variants |
| *Angiotensinogen (AGT)* | 183 | rs5049 | -217 | A/G | G | A |
| *Angiotensinogen (AGT)* | 183 | rs5051 | -6 | A/G | A | A |
| *G protein β3 subunit (GNB3)* | 2784 | rs5443 | 825 | C/T | C | T |
| *β2 adrenergic receptor (ADRB2)* | 154 | rs1042713 | 47 | A/G | G | 47A/79C |
| *β2 adrenergic receptor (ADRB2)* | 154 | rs1042714 | 79 | C/G | G |
| *Epithelial sodium channel α (ENaCα or SCNN1α)* | 6337 | rs3759324 | -946 | A/G | A | G |
| *Epithelial sodium channel γ (ENaCγ or SCNN1γ)* | 6340 | rs5718 | -173 | A/G | G | G |
