# Supplementary material for: Differential Susceptibility to Hypertension Is Due to Selection during the Out-of-Africa Expansion
Source: PLoS Genet. 2005 Dec 30;1(6):e82. doi: 10.1371/journal.pgen.0010082 (PMC1342636; doi:10.1371/journal.pgen.0010082)
Supplement: Table S2 — (91 KB DOC) [file pgen.0010082.st002.doc]

| Table S2: Population averages of phenotype and allele frequency data among the 35 INTERSALT populations with estimated *GNB3 825T* allele frequency. | | | | | | | | | | | |
| --- | --- | --- | --- | --- | --- | --- | --- | --- | --- | --- | --- |
| Population | Country | Latitude | Longitude | SBP (mmHg) | DBP (mmHg) | BMI (Kg/m^2^) | 24h Na (mmol/24 h) | Alcohol (ml/wk) | *GNB3 825T* (%) | N* | Source |
| Charleroi | Belgium | 50.42 | 4.43 | 125.1 | 77.8 | 25.9 | 141.2 | 94.3 | 30.8 | 1512 | [1] |
| Ghent | Belgium | 51.05 | 3.72 | 123.3 | 74.1 | 24.9 | 147.7 | 121.2 | 30.8 | 1512 |
| Xingu | Brazil | -7.73 | -68.72 | 99.8 | 62.2 | 23.4 | 12.3 | 0 | 26.0 | 25 | [2] |
| Yanomami | Brazil | 3.13 | 60.00 | 96.0 | 60.6 | 21.2 | 0.9 | 0 | 32.0 | 25 | [2] |
| Joensuu | Finland | 61.57 | 29.67 | 121.0 | 75.4 | 25.4 | 170.4 | 78.5 | 24.0 | 100 | [2] |
| Turku | Finland | 60.45 | 22.28 | 127.0 | 78.4 | 25.3 | 154.8 | 110.7 | 24.0 | 100 |
| Porcsalma Village | Hungary | 47.88 | 22.57 | 125.7 | 80.3 | 26.2 | 198.3 | 92.8 | 32.0 | 27 | [2] |
| New Delhi | India | 28.60 | 77.20 | 113.7 | 73.9 | 23.7 | 160.6 | 11.6 | 34.0 | 240 | [2] |
| Bassiano | Italy | 41.55 | 13.04 | 125.1 | 79.6 | 28.0 | 184.9 | 300.5 | 30.0 | 39 | [2,3] |
| Gubbio | Italy | 43.35 | 12.58 | 117.5 | 69.9 | 25.4 | 175.4 | 167.6 | 30.0 | 29 |
| Mirano | Italy | 45.49 | 12.10 | 119.4 | 76.0 | 25.4 | 174.1 | 228.9 | 32.0 | 45 | [3] |
| Osaka | Japan | 35.95 | 137.27 | 116.5 | 68.6 | 21.6 | 168.3 | 105.1 | 52.4 | 1640 | [2–7] |
| Tochigi Perfecture | Japan | 36.38 | 139.73 | 117.9 | 68.4 | 22.5 | 180.4 | 156.0 | 52.4 | 1640 |
| Toyama | Japan | 36.68 | 137.22 | 117.3 | 72.1 | 23.1 | 212.4 | 120.4 | 52.4 | 1640 |
| Asaro Valley | Papua NG | -6.58 | 145.98 | 108.0 | 62.7 | 21.7 | 36.8 | 7.9 | 56.4 | 47 | [2,3] |
| Beijing | China | 39.87 | 123.88 | 109.4 | 67.6 | 22.8 | 204.1 | 17.8 | 51.3 | 625 | [2,3,8] |
| Tianjin | China | 39.13 | 117.20 | 119.4 | 67.9 | 23.9 | 245.6 | 71.0 | 51.3 | 625 |
| Nanning | China | 22.82 | 108.32 | 110.7 | 70.5 | 21.3 | 169.2 | 11.8 | 43.0 | 244 | [2,3] |
| Krakow | Poland | 50.08 | 19.917 | 123.3 | 76.3 | 26.4 | 197.7 | 68.2 | 22.0 | 60 | [2] |
| Warsaw | Poland | 52.25 | 21.00 | 123.3 | 77.1 | 26.5 | 181.3 | 45.6 | 22.0 | 60 |
| Pusan | South Korea | 35.70 | 128.03 | 112.2 | 71.9 | 22.2 | 208.2 | 26.3 | 44.0 | 31 | [2] |
| Moscow | Soviet Union | 55.75 | 37.58 | 117.7 | 73.4 | 25.7 | 161.7 | 21.7 | 22.0 | 53 | [2,3] |
| Manresa | Spain | 41.73 | 1.83 | 119.7 | 71.8 | 25.4 | 174.6 | 160.0 | 30.0 | 30 | [2] |
| Torrejon | Spain | 40.65 | -3.33 | 119.9 | 68.6 | 26.7 | 183.2 | 164.3 | 30.0 | 30 |
| San Chilo village | Taiwan | 23.35 | 120.23 | 116.4 | 76.2 | 23.1 | 141.4 | 29.4 | 54.3 | 199 | [9] |
| Plymouth-Bethesada | Trinadad and Tobago | 11.22 | -60.78 | 118.3 | 75.0 | 28.2 | 117.4 | 73.8 | 85.0 | 10 | [2] |
| Belfast | United Kingdom | 54.58 | -5.93 | 120.3 | 73.8 | 24.8 | 150.8 | 229.7 | 30.0 | 163 | [10] |
| Goodman | US African-American | 32.58 | -89.54 | 119.5 | 76.4 | 30.3 | 103.6 | 190.1 | 74.0 | 821 | [2,11–13] |
| Jackson | US African-American | 32.32 | -90.21 | 125.6 | 71.5 | 28.0 | 150.9 | 59.6 | 74.0 | 821 |
| Goodman | US white | 32.58 | -89.54 | 114.2 | 79.2 | 28.2 | 130.8 | 45.6 | 29.1 | 1302 | [12,13] |
| Jackson | US white | 32.32 | -90.21 | 122.2 | 76.4 | 25.1 | 141.4 | 73.2 | 29.1 | 1302 |
| Cottbus | East Germany | 51.67 | 14.33 | 121.8 | 75.0 | 24.9 | 147.7 | 162.0 | 30.0 | 1855 | [2] |
| Bernried | West Germany | 49.32 | 12.55 | 122.8 | 74.9 | 24.5 | 167.0 | 135.7 | 30.0 | 1855 |
| Heidelberg | West Germany | 49.42 | 8.70 | 117.6 | 73.6 | 24.5 | 172.9 | 146.4 | 30.0 | 1855 |
| Harare | Zimbabwe | -17.86 | 31.03 | 123.7 | 76.8 | 26.1 | 140.5 | 171.7 | 81.0 | 299 | [2] |
| * Number of genotypes used to estimate population allele frequency | | | | | | | | | | | |

Table S2 Reference List

1. Brand E, Wang JG, Herrmann SM, Staessen JA (2003) An epidemiological study of blood pressure and metabolic phenotypes in relation to the G beta(3) C825T polymorphism. Journal of Hypertension 21: 729-737.

2. Siffert W, Forster P, Jockel KH, Mvere DA, Brinkmann B, et al. (1999) Worldwide ethnic distribution of the G protein beta3 subunit 825T allele and its association with obesity in Caucasian, Chinese, and Black African individuals. J Am Soc Nephrol 10: 1921-1930.

3. Cann HM, de Toma C, Cazes L, Legrand MF, Morel V, et al. (2002) A human genome diversity cell line panel. Science 296: 261-262.

4. Ishikawa K, Imai Y, Katsuya T, Ohkubo T, Tsuji I, et al. (2000) Human G-protein beta3 subunit variant is associated with serum potassium and total cholesterol levels but not with blood pressure. Am J Hypertens 13: 140-145.

5. Tozawa Y (2001) G protein beta3 subunit variant: tendency of increasing susceptibility to hypertension in Japanese. Blood Press 10: 131-134.

6. Kato N, Sugiyama T, Morita H, Kurihara H, Yamori Y, et al. (1998) G protein beta3 subunit variant and essential hypertension in Japanese. Hypertension 32: 935-938.

7. Tabara Y, Kohara K, Miki T (2002) Polymorphisms of genes encoding components of the sympathetic nervous system but not the renin-angiotensin system as risk factors for orthostatic hypotension. J Hypertens 20: 651-656.

8. Huang XH, Ju ZY, Song Y, Zhang HY, Sun K, et al. (2003) Lack of association between the G protein beta(3) subunit gene and essential hypertension in Chinese: a case-control and a family-based study. Journal of Molecular Medicine-Jmm 81: 729-735.

9. Tsai CH, Yeh HI, Chou Y, Liu HF, Yang TY, et al. (2000) G protein beta3 subunit variant and essential hypertension in Taiwan - a case-control study. Int J Cardiol 73: 191-195.

10. Brand E, Herrmann SM, Nicaud V, Ruidavets JB, Evans A, et al. (1999) The 825C/T polymorphism of the G-protein subunit beta3 is not related to hypertension. Hypertension 33: 1175-1178.

11. Larson N, Hutchinson R, Boerwinkle E (2000) Lack of association of 3 functional gene variants with hypertension in African Americans. Hypertension 35: 1297-1300.

12. Rankinen T, Rice T, Leon AS, Skinner JS, Wilmore JH, et al. (2002) G protein beta 3 polymorphism and hemodynamic and body composition phenotypes in the HERITAGE Family Study. Physiol Genomics 8: 151-157.

13. Morrison AC, Doris PA, Folsom AR, Nieto FJ, Boerwinkle E (2001) G-protein beta3 subunit and alpha-adducin polymorphisms and risk of subclinical and clinical stroke. Stroke 32: 822-829.
