# Supplementary material for: Differential Susceptibility to Hypertension Is Due to Selection during the Out-of-Africa Expansion
Source: PLoS Genet. 2005 Dec 30;1(6):e82. doi: 10.1371/journal.pgen.0010082 (PMC1342636; doi:10.1371/journal.pgen.0010082)
Supplement: Table S3 — (30 KB DOC) [file pgen.0010082.st003.doc]

| Table S3: Univariate models describing the association of traditional and nontraditional risk factors with population-average systolic blood pressure (mmHg) among 35 INTERSALT populations with genotype data. | | | | |
| --- | --- | --- | --- | --- |
|  | β | 95% CI | P | R^2^ |
| Traditional: |  |  |  |  |
| 24h Na | 0.08 | 0.04 – 0.12 | <.001 | 0.36 |
| BMI | 1.89 | 1.0 – 2.79 | <.001 | 0.36 |
| Alcohol | 0.05 | 0.02 – 0.08 | .001 | 0.30 |
| Nontraditional: |  |  |  |  |
| Absolute latitude | 0.31 | 0.20 – 0.43 | <.001 | 0.47 |
| *GNB3 825T* | -0.01 | -0.15 – 0.13 | .88 | 0.00 |
